# Longitudinal analysis of physical activity, sedentary behaviour and anthropometric measures from ages 6 to 11 years

**DOI:** 10.1186/s12966-018-0756-3

**Published:** 2018-12-07

**Authors:** Phillipp Schwarzfischer, Dariusz Gruszfeld, Piotr Socha, Veronica Luque, Ricardo Closa-Monasterolo, Déborah Rousseaux, Melissa Moretti, Benedetta Mariani, Elvira Verduci, Berthold Koletzko, Veit Grote

**Affiliations:** 1 Division of Metabolic and Nutritional Medicine, LMU - Ludwig-Maximilians-Universität München, Dr. von Hauner Children’s Hospital, Lindwurmstr 4, 80337 Muenchen, Germany; 2 Neonatal Intensive Care Unit, Children’s Memorial Health Institute, Warsaw, Poland; 3 Department of Gastroenterology, Children’s Memorial Health Institute, Warsaw, Poland; 4 Paediatrics Research Unit, Universitat Rovira i Virgili, IISPV, Reus, Spain; 5 CHC St. Vincent, Liège-Rocourt, Belgium; 6 Department of Paediatrics, San Paolo Hospital, University of Milan, Milan, Italy

**Keywords:** Obesity, Accelerometer, *SenseWear* armband, Light physical activity, MVPA, CHOP

## Abstract

**Background/Objectives:**

The aim of this study was to examine the effect of physical activity (PA) and sedentary behaviour (SB) on body mass index (BMI) and fat mass index (FMI) in children over the course of five years and identify potential bi-directional associations.

**Subjects/Methods:**

Data were drawn from the EU Childhood Obesity Project (CHOP). PA and SB were measured with the *SenseWear* Armband 2 at the ages of 6 (T1), 8 (T2) and 11 (T3) years. Height and weight were measured and BMI was calculated at each time point, resulting in 1254 complete observations from 600 children. Bio impedance analysis was used to measure body fat mass and eventually calculate FMI. To examine the longitudinal association between PA/SB and BMI/FMI as well as to account for repeated measure on these children, mixed model analysis was employed.

**Results:**

Higher levels of total PA and moderate-to-vigorous PA (MVPA) were associated with lower BMI and FMI and higher SB with higher BMI and FMI over the five year period. When looking at the age dependent effects, negative associations of MVPA (β_MVPA x age_: − 0.05, 95% confidence interval (CI): − 0.09 – -0.01, *p* = 0.007) and positive associations of SB (β_SB x age_: 0.04, 95% CI: 0.02–0.06, *p* < 0.001) increased with each year of age. In a model combining these two effects, only SB x age interaction remained significant (β_SB x age_: 0.04, 95% CI: 0.03–0.06, *p* = 0.01). No significant interaction between MVPA and SB could be discerned. Light Physical activity showed no significant associations with BMI or FMI. When reversing outcome and predictor; higher BMI or FMI showed a negative association with MVPA and a positive association with SB, but no age dependency.

**Conclusions:**

More time per day in SB was associated with a higher BMI over the course of five years, whereas higher MVPA had an inverse effect. In a combined model, only effects of higher SB remained significant, emphasizing the importance of SB in obesity prevention. Present bidirectional associations, where lower body size was associated with higher PA and lower SB, indicated the need for an integrated approach of activity and weight control for obesity prevention.

**Trial registration:**

ClinicalTrials.gov Identifier: NCT00338689. Registered: June 19, 2006 (retrospectively registered).

**Electronic supplementary material:**

The online version of this article (10.1186/s12966-018-0756-3) contains supplementary material, which is available to authorized users.

## Introduction

The increase of childhood obesity is a major public health problem in Europe and other affluent countries [1–3]. Changes in childhood movement behaviour might play an important role in childhood obesity risk. High levels of physical activity (PA) are thought to be protective against excess body weight [4]. A recent systematic review showed that sedentary behaviour (SB) is related to many adverse health indicators, including unfavourable body composition [5]. Many of the studies included in the systematic review used subjective methods for measuring SB. Subjective methods can be a good estimate for context-specific SB like screen time, however, device-based methods can provide a more reliable and valid assessment of overall SB [6].

Overall the associations of PA and SB with obesity are inconsistent [7–10] and there is a lack of high quality studies examining the combined effect of device-based measurements of SB and PA on anthropometric measures. It remains unclear if positive health effects of moderate-to-vigorous PA (MVPA) outweigh negative health effects of high SB. Additionally, the direction of the effects need further clarification, as reversed causality could not be ruled out [11]. Evidence for reverse causality was found in a longitudinal observational analysis of 785 children, with a follow-up of 200 days and device-based measurement of PA [12]. In that study, a higher fat mass index (FMI) at baseline was associated with lower PA and more SB, whereas baseline movement behaviour did not predict any subsequent change of FMI. The results of other prospective observational studies employing device-based PA measurement found a bi-directional association [13] or no association in either direction [14]. Therefore, there is a need for more analyses of bi-directional associations.

This study is a secondary analysis of data from the EU Childhood Obesity Project (CHOP) a multicentre, randomized intervention trial taking place in five European countries. The current study may help to better understand the interplay of PA and SB in development of excess weight, by employing device-based measurement of PA and SB in a long-term cohort study. The primary aim is to examine associations between PA, light PA (LPA), MVPA, SB and the development of body mass index (BMI) and fat mass index (FMI) from 6 to 11 years of age. The secondary aim is to test for potential bi-directional effects of associations.

## Methods

### Study subjects and design

The CHOP study was initially started in 2002 and recruited 1678 infants in Europe during the first 8 weeks of life. This randomized control trial (clinical trial registry: NCT00338689) investigated the influence of higher and lower protein content infant formula during the first year of life on length and weight gain during infancy and childhood. Besides those two intervention groups the study also included an observational group of breastfed children. Its design and outcomes are reported elsewhere [15, 16]. Data for this analysis were collected during the 6- (T1), 8- (T2) and 11-year (T3) follow-up examinations. Sample collection was coordinated by 5 study teams in 8 municipalities in Germany (Nuremberg, Munich), Italy (Milano), Belgium (Brussels, Liege), Poland (Warsaw) and Spain (Reus, Tarragona). The trial was approved by ethics committees in each study centre and informed consent was obtained by parents. All research was performed in accordance with the Declaration of Helsinki.

### Activity assessment

At each of the three study visits, parents and children participated in accelerometer measurements. SB and PA levels were measured using the *SenseWear* Armband 2 (Body Media Inc., Pittsburgh, PA, USA). This device is worn over the right triceps muscle and incorporates five sensors: two-axis accelerometer (for movement patterns and step-count), galvanic skin response, skin temperature, near body temperature sensor and heat flux [17]. Recording epoch length were 1 min intervals. Children wore the armband day and night on 3 consecutive days, including one weekday and one weekend day. Valid days were defined as at least 20 h·day^− 1^ of recording. This time frame was proposed by Trost et al. [18] for accelerometer based studies. Observations are defined as one accelerometer measurement of each child at one of the three time points. Observations with only one day of recording were excluded. Two-day observations showed no differences to 3-day observations and were included in the analysis. Additionally, only observations with at least one weekday and one weekend day were included.

Data from armbands were processed with the Professional *InnerView* Software 6.1 (Body Media Inc., Pittsburgh, PA) already described elsewhere [19]. Intensity levels of PA were classified by Metabolic Equivalents of Task (METs): LPA 1.5–3.9 METs and MVPA ≥4 METs [20], were total PA included both LPA and MVPA minutes (i.e. > 1.5 METs). Awake minutes below 1.5 METs were seen as SB, in accordance to the cut-off set by the Sedentary Behavior Research Network [21, 22]. This definition additional includes the posture component of SB, i.e. sitting and reclined positions. The *SenseWear* Armband 2 cannot differ between standing and sitting and no direct observations or questionnaire data about activities were available. However, the armband was validated against a posture measuring device in children (11–13 year old) and were found to be accurate in measuring resting activities [23]. Other validation studies in children showed that the *SenseWear InnerView* algorithms (version 6.1) used in our study produce valid estimates of energy expenditure for assessing PA and SB in children [24, 25].

### Anthropometry

During each follow up visit weight and height measurements were taken. The same scale (SECA 702, seca gmbh & co. kg., Hamburg, Germany) for weight and the same stadiometer (SECA 242, seca gmbh & co. kg., Hamburg, Germany) for height were used in each site. Standard operating procedures relied on the World Health Organisation’s Growth Reference Study [26]. BMI (weight [kg]/height [m]^2^) was calculated. Body fat mass was calculated from bioelectrical impedance assessed in duplicate with the octopolar *Tanita BC-418* (Tanita Corporation, Tokyo, Japan). FMI was calculated (total body fat mass [kg]/height [m]^2^). Measurement with the *Tanita BC-418* was validated for use in 7 year old children from our sample [27]. It was seen that the device can give precise measurements to estimate children’s body composition in an epidemiological setting, but should be treated with caution at an individual level.

### Covariates

Gender, intervention group (higher and lower protein infant formula) or breasted group, wear time of device per day and study country data were available for all children. Additional known risk factors for childhood obesity with potential effect on PA and SB were available. This includes birth weight of the child, which was measured by study nurses right after birth or retrieved from medical records. Caloric intake (kcal) was assessed at each time point. While 3-day food protocols were used at T1 and T2, a food frequency questionnaire was used at T3. To facilitate the analysis of this data, tertiles were formed, which represent low, mid and high caloric intake at each time point. At 11 years puberty status was assessed with the ‘Pubertal Development Scale’ and categorised as ‘pre-pubertal’ and ‘pubertal’ [28].

### Data analyses

Data are reported as mean with standard deviation (SD) for continuous variables and as number (n) and percentage (%) for categorical variables. Mixed models with a random individual intercept and random slope over age were used with either BMI or FMI at T1, T2 and T3 as time variant outcomes. Primarily, the effects of SB, total PA, MVPA and LPA on respective outcomes were modelled separately. We scaled all activities to 60 min/day to ensure meaningful effect sizes and make results of each model comparable. Gender, intervention group and study country were included in all models and additional covariates (birthweight and caloric intake) were added separately and kept for all following models upon improving overall model fit. In a second step, age interactions for each significant main predictors (PA x age, MVPA x age, LPA x age and SB x age) were added, to test for potential age dependent effects. As a last step, MVPA and SB (and their respective interaction with age) were jointly included in one model to test mutual adjusted main effects (Model 1) and age dependent associations (Model 2). To avoid collinearity, we looked at correlations between total PA, SB and MVPA at each time point. Additionally, interaction between SB and MVPA was tested (Model 3). Age was centred to the lowest age of participants, 5.89 years. To test if associations in a mutual adjusted model are moderated by either SB or MVPA, models were replicated only in children with high MVPA or high SB. High MVPA and SB was defined by the highest tertile of the average time in MVPA and SB over the 5 year period (all 3 measurement points). To examine possible bi-directional effects, mixed effects model outcomes (FMI and BMI) and main predictors (SB and MVPA) were reversed and adjusted for age, age^2^ (as both SB and MVPA showed a quadratic development with age), gender and country. Interaction of FMI and BMI with age (BMI x age and FMI x age) and other covariates were tested in both models. In FMI and MVPA models, outcomes were log transformed for analysis, due to skewness of residuals. For interpretation, log transformed values were later back transformed. All models were optimized by maximum likelihood estimation and likelihood-ratio tests were used to test for the best model fit.

To assess the influence of missing data, we ran two sensitivity analyses, one with the sample restricted to those with 3 days of accelerometer recording per observation and one with the sample of children with two or more time points. Models were calculated in R using the ‘lme4’ package. Significance was assumed at an error probability < 0.05.

## Results

Due to loss to follow-up, the number of children attending follow up visits decreased from 661 children at T1 to 589 children at T2 and 583 children at T3. Participation rate of accelerometer measurement increased from 63.1% at T1 (417 of 661 children) to 70.5% at T2 (415 of 589 children) and 72.4% at T3 (422 of 583 children). In total, 600 children with complete data on BMI and accelerometer data were included in the analysis, resulting in 1254 observations (Table 1); 430 children had at least two measurements. FMI was missing in 47 observations, resulting in 1207 valid observations of 586 children. There were no differences in anthropometric data between children, who participated in accelerometer measurement (*n* = 600) and those who did not (*n* = 126).

**Table 1:** Characteristics and activity levels of participants for each time point

		6 years	8 years	11 years
n		417	415	422
Male	n (%)	184 (44.1%)	199 (48.0%)	191 (45.3%)
Age, years	mean (SD)	6.1 (0.1)	8.1 (0.1)	11.2 (0.2)
Anthropometry				
BMI	mean (SD)	15.9 (2.0)	16.8 (2.6)	18.7 (3.3)
FMI	mean (SD)	3.4 (1.1)	3.9 (1.6)	4.5 (2.0)
Activity levels in minutes per day				
Sedentary	mean (SD)	299.0 (79.6)	332.0 (79.9)	406.0 (96.7)
PA	mean (SD)	532.9 (82.3)	519.8 (80.4)	457.6 (100.6)
Light PA	mean (SD)	418.3 (69.7)	397.8 (71.8)	373.7 (81.1)
MVPA	mean (SD)	114.6 (59.5)	122.1 (72.3)	83.9 (53.6)

*Abbreviations:* *BMI* Body mass index, *FMI* Fat mass index, *SB* Sedentary behaviour, *PA* Physical activity, *MVPA* Moderate-to-vigorous physical activity

Table 2 shows the effects of SB, total PA, MVPA and LPA in a mixed model analysis with either BMI or FMI as outcome. In summary, more time spent per day in SB was associated with a 0.13 kg/m^2^ higher BMI (*p* < 0.001) and 0.05 kg/m^2^ higher FMI (*p* < 0.001), whereas PA levels showed inverse associations. Each additional hour in total PA was associated with a − 0.11 kg/m^2^ reduced BMI (p < 0.001) and a − 0.03 kg/m^2^ reduced FMI (*p* = 0.001). Similar results were seen for MVPA with BMI and FMI with slightly lager effect sizes. Time in LPA showed no significant results. Adding caloric intake and puberty status showed no improvement of model fit. Birthweight improved model fit of BMI models and thus was included in all BMI outcome models.

**Table 2:** Mixed model estimates of the association between SB and PA levels and anthropometric measures

		BMI			FMI	
	β	95% CI		β	95% CI	
SB	0.13	0.07–0.19	***	0.05	0.03–0.06	***
Total PA	−0.11	− 0.17 – − 0.05	***	− 0.03	− 0.06 – − 0.01	**
MVPA	− 0.14	−0.21 – − 0.08	***	− 0.05	−0.08 – − 0.03	***
LPA	0.00	−0.07 – 0.06		0.00	0.03 – −0.03	

*Abbreviations:* *SB* Sedentary behaviour per 60 min/day, *Total PA* Total physical activity per 60 min/day, *MVPA* Moderate-to-vigorous PA per 60 min/day, *LPA* Light PA in 60 min/day, *BMI* Body mass index (kg/m^2^), *FMI* Fat mass index(kg/m^2^), *CI* Confidence interval

Data were analysed with the use of 8 separate mixed models, adjusted for covariates intervention group, gender, wear time and country

*P < 0.05, **P < 0.01, ***P < 0.001

Table 3 shows the age dependent associations between time in PA levels, SB and anthropometric measures. An additional 60 min/day of MVPA were associated with an 0.05 kg/m^2^ lower BMI per year (*p* = 0.006) and 60 min more SB per day were associated with a 0.04 kg/m^2^ higher BMI per year (*p* < 0.001). Interaction between total PA and age was not significant (*p* = 0.080). Association of total PA, MVPA and FMI did not significantly differ with age.

**Table 3:** Age dependent associations of time spent in sedentary behaviour, total and moderate-to-vigorous physical activity and anthropometric measures

		SB model			PA model			MVPA model	
Outcome: Body mass index									
	β	95% CI		β	95% CI		β	95% CI	
Age	0.26	0.13–0.39	***	0.66	0.48–0.84	***	0.60	0.53–0.67	***
SB	0.04	−0.04 – 0.11							
SB x Age	0.04	0.02–0.06	***						
Total PA				−0.06	−0.14 – 0.01				
Total PA x Age				−0.02	−0.04 – 0.00				
MVPA							−0.04	−0.14 – 0.05	
MVPA x Age							−0.05	−0.09 – − 0.01	**
Outcome: Fat mass index									
Age	0.10	0.03–0.23	***	0.08	0.02–0.21	**	0.11	0.06–0.21	***
SB	0.05	0.02–0.09	**						
SB x Age	0.00	0.00–0.00							
Total PA				−0.05	−0.05 – 0.00	**			
Total PA x Age				0.00	0.00–0.00				
MVPA							−0.03	− 0.05 – 0.00	
MVPA x Age							0.00	−0.03 – 0.00	

*Abbreviations:* *SB* sedentary behaviour per 60 min/day, *Total PA* total physical activity per 60 min/day, *MVPA* moderate to vigorous PA per 60 min/day, *CI* confidence interval *P < 0.05, **P < 0.01, ***P < 0.001

Data were analysed with the use of 3 separate mixed models, adjusted for covariates gender, intervention group, wear time and study country; Age was centred to the lowest age of any participant (5.89 years).

Total PA and SB were highly correlated at all time points (*r* > 0.75) and were not included in a combined model. As correlation between MVPA and SB was low to medium at each time point (T1: *r* = − 0.45, *p* < 0.001; T2: r = − 0.45, *p* < 0.001; T3: *r* = − 0.55, *p* < 0.001) we included both in a combined model. Table 4 shows the results of this joint analysis. Model 1 shows the main effects. Both MVPA and SB remained significant with similar effects sizes in opposite directions (β_MVPA_: -0.10, 95% CI: -0.17 – -0.02, *p* = 0.014; β_SB_: 0.09, 95% CI: 0.03–0.16, *p* = 0.013). When including age interactions in model 2, only SB x age remained significant (β_SB_: 0.01, 95% CI: -0.08 – 0.10, *p* = 0.824; β_SB x age_: 0.03, 95% CI: 0.01–0.05, *p* = 0.012). Testing for an interaction between SB and MVPA showed a negative association with BMI, but was not significant (β_SB x MVPA_: -0.02, 95% CI: -0.06 – 0.02, *p* = 0.275; Table 4, Model 3). Analysis of effects of SB and SB age interaction in a sample of children with high MVPA can be found in Additional file 1: Table S1. In summary moderating effects of MVPA can be ruled out, even though significance for the SB x age interactions were lost (β_SB x age (high MVPA)_: 0.03, 95% CI: -0.00 – 0.06, *p* = 0.074). This was probably caused by loss of power (*n* = 200) due to splitting the sample in tertiles.

**Table 4:** Combined main effects (Model 1) and age dependent effects (Model 2) and interaction (Model 3) of time spent in sedentary behaviour and moderate-to-vigorous physical activity on body mass index

	Model 1			Model 2			Model 3		
	β	95% CI		β	95% CI		β	95% CI	
Age	0.49	0.45–0.54	***	0.33	0.13–0.52	**	0.49	0.45–0.54	***
SB	0.09	0.03–0.16	**	0.01	−0.08 – 0.10		0.12	0.03–0.21	*
MVPA	−0.10	−0.17 – − 0.02	*	−0.06	− 0.17 – 0.05		0.01	− 0.20 – 0.22	
SB x Age				0.03	0.01–0.06	*			
MVPA x Age				−0.02	−0.06 – 0.03				
SB x MVPA							−0.02	−0.06 – 0.02	

*Abbreviations:* *SB* sedentary behaviour per 60 min/day, *MVPA* moderate to vigorous PA per 60 min/day, *CI* confidence interval

Data were analysed with the use of 3 separate mixed models, adjusted for covariates gender, intervention group, wear time and study country; age was centred to the lowest age of any participant (5.89 years)

*P < 0.05, **P < 0.01, ***P < 0.001

When reversing outcome and predictor, higher FMI and BMI were associated with higher levels of SB (β_BMI_: 6.26, 95% CI: 4.27–8.25, *p* < 0.001; β_FMI_: 12.05, 95% CI: 8.64–15.47, *p* < 0.001), but no significant age interactions (BMI x age, FMI x age) were found. Similar results were seen for MVPA outcome models (β_BMI_: -7.61, 95% CI: -8.07 – -3.04, *p* < 0.001; β_FMI_: -9.09, 95% CI: -11.95 – -7.14, *p* < 0.001), with no significant age interactions.

Sensitivity analysis on the per-protocol subsample with 3 days of recording (1090 observations, 566 children) was performed with all models. Significance did not change in all BMI and FMI models and only slight changes in estimates were seen. Further analyses were repeated with children who participated at least 2 time points (1084 observations, 430 children) and similar results were achieved with only slight changes of estimates and associations remaining significant.

## Discussion

### Main study findings and implications

In this study, more time spent in SB was consistently associated with higher BMI. In a mutually-adjusted model, effects sizes of SB and MVPA were of equal magnitude but in opposite directions. When testing age interactions, only associations between SB and BMI remained significant. Further analysis showed that the positive association between SB and BMI increased with age, whereas BMI showed a stable association with SB over time, regardless of age. Overall, our results suggest that SB may play an important role in childhood overweight and obesity development.

The study confirms emerging evidence of a negative association between SB in childhood (device-based measurements) with BMI, even when concurrent levels of MVPA are considered. A study by Mitchell et al. [29] also used device-based measurement methods in 789 children between 9 and 15 years of age. Over the observed age period, SB was associated with an increasing BMI in children on the 50th, 75th and 90th BMI percentiles, when applying quantile regression. Another recent study by Mann et al. [30] employed bivariate linear spline models to test the independent effect of SB on adiposity markers in children 7 to 15 years of age, after adjustment for MVPA. Increasing SB was associated with an annual increased BMI (0.07 kg/m^2^, 95% CI: 0.06–0.09) and annual increased FMI (0.14 kg/m^2^, 95% CI: 0.10–0.18). However, the reported associations between SB and obesity indices are not uniform. Another international cross-sectional study in a sample of 6539 children examined the relationship between MVPA, SB and obesity. It found that only MVPA, but not SB, was significantly associated with BMI in a mutually adjusted model [31]. Thus, the interplay of higher SB, low PA and obesity needs further clarification. Long-term cohort studies in childhood (with multiple measurement points) would help to better understand the impact of levels and changes of PA and SB on later obesity risk.

In the present study, cross-sectional associations between movement behaviour and BMI as well as FMI were found, but no age interactions between movement behaviour and FMI were found. Basterfield et al. [32] reported that proxies for body weight (like BMI) are inferior to direct measures of body composition, when looking at adiposity outcomes. However, body fat mass from impedance measurements give fairly good body composition measures but lack precision [33], which might explain the lack of significant longitudinal effects of PA on FMI in our study. Additionally, effects sizes of associations with BMI in our study and other studies were rather small [12, 30, 34]. This brings to question, whether PA promotion is an adequate or effective tool for obesity prevention. Intervention studies are needed to clarify whether a substantial increase in habitual PA and a reduction of SB can result in a meaningful change of obesity markers in children.

In our study, results of an inverse analysis showed that higher BMI or FMI are associated with lower levels of MVPA and higher levels of SB, supporting the hypothesis of a bi-directional association. We also showed an age-dependent association between higher SB and higher BMI; higher BMI showed a stable association with higher SB over time. These results are similar to findings of a study from Marques et al. [9] where in a sample of 10- to 11-year old children a bi-directional association was only seen on cross-sectional, but not prospective analysis. These ambiguous results, taken together with inconsistent results from other studies [12–14], do not yet allow a firm conclusion about the direction of effects. Our study indicates that a consistently increased SB results in a higher BMI at later ages, whereas a consistently high BMI seems not to increase SB levels.

Our results stress the importance of reducing excess time in SB for the prevention of childhood obesity. Nevertheless, the practical application of our findings is difficult. In order to reduce SB, time spent in SB needs to be replaced by a form of PA, either LPA or MVPA. LPA comprises the majority of PA, about 80% of total PA measured in our cohort. Due to its light intensity, LPA is an “easy” substitute to SB. Effects of LPA on anthropometric measures range from showing a negative association with fat mass [35, 36], to no relationship, a finding reported in our study as well as other studies [37]. Thus, increasing LPA as a preventative measure for obesity is still unclear. Furthermore, interventions trying to increase children’s PA and MVPA lack convincing results [38] and show variable success rates for childhood obesity prevention [39, 40]. Additionally, when looking at effect sizes from our results and others [30, 37], even with substantial increases in MVPA, only small reductions of BMI could be achieved.

### Strengths and limitations

A strength of our study includes the longitudinal multicentre design of children born after the year 2000. Other strengths include device-based measurement of activity at each time point with high quality measurements of outcomes using standardised methods, with adjustment for various potential confounders.

In terms of generalizability of the findings, study participants were from Western European countries and mainly from metropolitan areas, making the results of this study generalizable to children with similar demographics. This was a secondary analysis of a randomized intervention trial whose a-priori hypothesis was the effect of varying protein content in infant formula on obesity risk. The intervention (high and low protein infant formula) showed an effect on BMI until 6 years of age [16], which might have influence the results. However, in this secondary analysis of childhood movement behaviour, the high and low protein intervention did not directly affect SB or PA levels. Adjustment for the intervention groups improved the overall fit of our statistical models. No confounding effects or interactions of early life intervention groups were observed.

PA and SB were measured at each of the three time points, which allowed for change in activity levels to be accounted for, over the observed time period, and allowed for modelling potential effects on anthropometric outcomes. Final models included age interactions for PA as well as SB, an aspect which other published studies are lacking [14, 34]. Accounting for possible age effects is of unique importance, since the period between childhood and adolescence is characterized by various changes in PA and SB [41]. The generation of children born after the year 2000 generally have a different lifestyle, compared to older generations, which is largely influenced by digitization of extracurricular activities. For example, universal access to telecommunication via mobile phones, use of smart phones, video games, time spent watching television or other ‘screen time’ activities, which influences PA and SB is significantly higher compared to older generations.

The results of our study are based on a European birth cohort and employed high quality measurement methods, which makes results generalizable to European children. However, some methodological factors, together with the relatively small sample size due to attrition, limit the external validity of our study to some extent.

Although device-based measurement of PA and SB can be a more reliable and more valid type of assessment of overall daily SB compared to self-report [6], it is difficult to compare results of accelerometer-based studies to other studies due to various differences. These differences include: cut-off values for intensities, epoch’s lengths, number of days measured and the various devices used for PA and SB measurement. The *SenseWear* armband provides many advantages over other instruments, since it is a combination of a conventional accelerometer but with additional body sensors. Nevertheless, it is rarely used in PA-related science. This makes our study difficult to directly compare to other studies which did not sure the *SenseWear* armband.

Another limitation of our measurement is the relatively short measurement period (3 days). Additionally, there was a lack of a wear-time protocol, which might have biased the activity measurement results. However, the identification of wear-time and non-wear-time was not an issue, as the *SenseWear* armband automatically detects when the device is taken off, due to its detection of body heat. Due to the detection of body heat, the advantage of measuring SB with the *SenseWear* device is that the number for minutes in SB is more reliably measures than with other devices. Most other accelerometer-based studies approximate non-wear-time by consecutive zeros in device outputs (non-wear criteria ranging from 20 min of consecutive zeros to 60 min with and without allowance of interruptions). Problems arose when identifying time spent sleeping. With an average of 7 h of sleep per day, daily sleeping time classified by the *Sensewear,* was relatively short. After adding lying time to time spent sleeping, a more realistic daily average was calculated, at about 9.2 h per day. Potential lying time or time spent in reclined positions during the day (which is normally defined as SB [22]) was excluded, resulting in a slight underestimation of SB. Additionally, validation studies in children found inaccurate measures of energy expenditure in children when using armbands, which subsequently lead to a misclassification of activities [42–44]. While the algorithm of the armband and its software has improved over the years, the latest versions were not available for our data [45].

## Conclusion

In summary, children that spent a longer time in SB had a higher BMI, even when adjusting for time spent in MVPA. This observation supports inactivity as an independent risk factor for childhood obesity. On the other hand, a shorter time spent in MVPA predicted a reduced BMI over a 5-year period. Effect sizes were rather small, however, and were no longer significant after adjustment for SB. In future interventions for obesity prevention, the focus shouldn’t solely be on increasing high intensity PA, but should also emphasise reducing time spent in SB. Although LPA showed no associations with BMI, promotion of LPA to reduce SB might be a more realistic target than promotion of MVPA alone. Lack of results regarding adiposity measures, like FMI, demonstrates the need for more studies examining the combined effects of SB and PA on obesity and adiposity.

## Additional file

Additional file 1: **Table S1.** Age dependent associations of time spent in sedentary behaviour and anthropometric measures in a subsample of children of the highest tertile of MVPA over the three measurement points (*n* = 200). (DOCX 13 kb)

## Abbreviations

CHOP: Childhood obesity project
CI: Confidence interval
FMI: Fat mass index
LPA: Light physical activity
MET: Metabolic equivalents of task
min: Minutes
MVPA: Moderate to vigorous physical activity
PA: Physical activity
SB: Sedentary behaviour
