# Correction to: longitudinal analysis of physical activity, sedentary behaviour and anthropometric measures from ages 6 to 11 years

**DOI:** 10.1186/s12966-019-0785-6

**Published:** 2019-03-04

**Authors:** Phillipp Schwarzfischer, Dariusz Gruszfeld, Piotr Socha, Veronica Luque, Ricardo Closa-Monasterolo, Déborah Rousseaux, Melissa Moretti, Benedetta Mariani, Elvira Verduci, Berthold Koletzko, Veit Grote

**Affiliations:** 1 Division of Metabolic and Nutritional Medicine, LMU - Ludwig-Maximilians-Universität München, Dr. von Hauner Children’s Hospital, Lindwurmstr 4, 80337 Muenchen, Germany; 2 Neonatal Intensive Care Unit, Children’s Memorial Health Institute, Warsaw, Poland; 3 Department of Gastroenterology, Children’s Memorial Health Institute, Warsaw, Poland; 4 Paediatrics Research Unit, Universitat Rovira i Virgili, IISPV, Reus, Spain; 5 CHC St. Vincent, Liège-Rocourt, Belgium; 6 Department of Paediatrics, San Paolo Hospital, University of Milan, Milan, Italy

**Correction to: International Journal of Behavioral Nutrition and Physical Activity (2018) 15:126**

**https://doi.org/10.1186/s12966-018-0756-3**

Following publication of the original article [1], the author reported an error in the ‘Conclusion’ section as follows:

Incorrect statement in published article: “On the other hand, a shorter time spent in MVPA predicted a **reduced** BMI over a 5-year period.”

Corrected statement: “On the other hand, a shorter time spent in MVPA predicted an **increased** BMI over a 5-year period.”

## References

[1] Schwarzfischer et al. Int J Behav Nutr Phys Act (2018) 15:126 doi 10.1186/s12966-018-0756-3 10.1186/s12966-018-0756-3 PMC6286599 30526600

