# Supplementary material for: Longitudinal analysis of physical activity, sedentary behaviour and anthropometric measures from ages 6 to 11 years
Source: Int J Behav Nutr Phys Act. 2018 Dec 7;15:126. doi: 10.1186/s12966-018-0756-3 (PMC6286599; doi:10.1186/s12966-018-0756-3)
Supplement: Supplementary file 1 — Table S1. Age dependent associations of time spent in sedentary behaviour and anthropometric measures in a subsample of children of the highest tertile of MVPA over the three measurement points (n = 200). (DOCX 13 kb) [file 12966_2018_756_MOESM1_ESM.docx]

Additional file 1

Table S1. Age dependent associations of time spent in sedentary behaviour and anthropometric measures in a subsample of children of the highest tertile of MVPA over the three measurement points (n = 200).

|  |  | BMI |  |  | BMI |  |
| --- | --- | --- | --- | --- | --- | --- |
|  | β | 95% CI | p-value | β | 95% CI | p-value |
| Age | 0.46 | 0.40 – 0.51 | **<.001** | 0.3 | 0.12 – 0.47 | **0.001** |
| SB | 0.09 | 0.01 – 0.17 | **0.026** | 0.02 | -0.09 – 0.13 | 0.746 |
| SB x Age |  |  |  | 0.03 | -0.00 – 0.06 | 0.074 |

*Data were analysed with the use of 2 separate mixed models, adjusted for covariates gender, intervention group, wear time and study country; age was centred to the lowest age of any participant (5.89 years).*

*Abbreviations: SB, sedentary behaviour per 60 min/day; CI, confidence interval*
